# Impact of albendazole treatment on the symptom profile of neurocysticercosis patients 14–16 years following diagnosis

**DOI:** 10.1017/S003118202500023X

**Published:** 2025-07

**Authors:** Lucila Vilela, Zachary Shahn, Arturo Carpio, W. Luis Yepez, Daniela Di Capua, Alex Jaramillo, W. Allen Hauser, Karina Quinde-Herrera, Elizabeth A. Kelvin

**Affiliations:** 1 Department of Epidemiology and Biostatistics, CUNY Graduate School of Public Health and Health Policy, City University of New York, New York, NY, USA; 2 CUNY Institute for Implementation Science in Population Health, City University of New York, New York, NY, USA; 3 School of Medicine, University of Cuenca, Cuenca, Ecuador; 4 Hospital Los Ceibos de Guayaquil, Ecuador; 5 Hospital Eugenio Espejo, Ministerio de Salud Pública, Quito, Ecuador; 6 Universidad San Francisco de Quito, Ecuador; 7 Instituto de Diagnóstico por Imágenes, Cuenca, Ecuador; 8 Gertrude H. Sergievsky Center, Columbia University, New York, NY, USA; 9 Donald and Barbara Zucker School of Medicine at Hofstra University/Northwell Health, Hempstead, NY, USA

**Keywords:** Ecuador, neurocysticercosis, randomized controlled trial, seizures, symptoms, *Taenia solium*

## Abstract

Neurocysticercosis (NCC) is a neglected parasitic disease that causes neurological symptoms. However, little is known about the long-term impact of this infection on health. We contacted participants from a randomized controlled trial on albendazole treatment for NCC in Ecuador 12 years after trial completion (14–16 years after NCC diagnosis) about their long-term health. We described the symptoms experienced post-trial and investigated if albendazole treatment, the presence of calcified NC cysts, and cysts in extraparenchymal locations at last imaging predicted symptoms. All analyses were standardized by adjusting for participant age and sex. In the 12 years post-trial, 52.1% reported some health problem, with 48.9% reporting neurological symptoms such as seizures (16.6% of participants) and headaches (26.6% of participants). At the end of the trial, 11 participants had complete NCC cyst resolution, of whom 3 (27.3%) reported seizures and 1 (9.1%) reported headaches post-trial. Twenty-four participants had only calcified cysts (residual calcification sometimes left after the parasite dies) by trial end, of whom 8 (33.3%) reported seizures and 9 (37.5%) headaches post-trial. None of the predictors examined were significantly associated with long-term symptoms. A high proportion of people diagnosed with NCC continue experiencing symptoms years after treatment, and while slightly fewer people experienced continued symptoms in the albendazole group, the difference was not statistically significant. Eleven participants with no live parasites at last imaging (8 with residual calcifications) had seizures post-trial, which may be unprovoked and an indication of epilepsy risk. Research is urgently needed to improve NCC treatment to mitigate long-term outcomes.

## Graphical abstract

**Figure:**
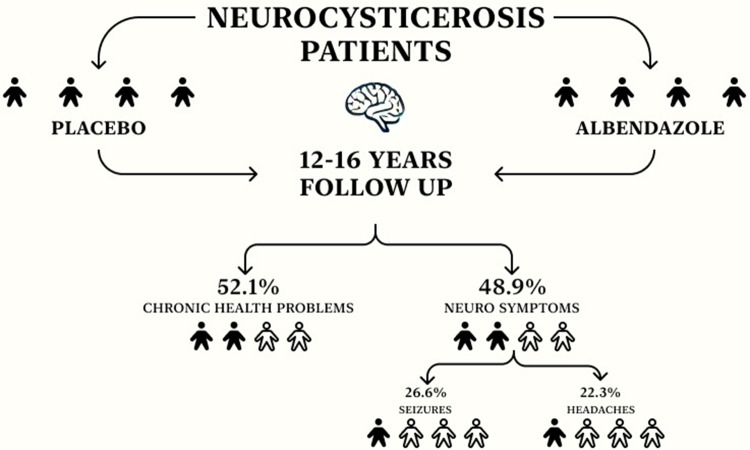

## Introduction

Neurocysticercosis (NCC) is an infection of the central nervous system (CNS) with the larval form of the pork tapeworm, *Taenia solium (T. solium*), which occurs when humans ingest the parasite eggs, usually by consuming contaminated food or water. In humans, *T. solium* larvae are commonly found in CNS tissue (Fleury et al., 2004). NCC is the most common parasitic disease affecting the CNS and an important cause of seizures in low- and middle-income countries. NCC diagnosis also occurs in high-income countries (Wallin and Kurtzke, 2004), likely due to international travel and immigration (O’Neal and Flecker, 2015), although local transmission within the United States (US) has been documented (CDC, 1992; Schantz et al., 1992). The World Health Organization estimates that the total number of people infected with NCC is 2.56–8.30 million (WHO, 2022b), with 50 000 deaths from NCC occurring every year (Savioli, 2010). Symptoms, including seizures, result in an estimated 2.8 million disability-adjusted life-years lost (WHO, 2022b). NCC also causes economic losses due to the cost of diagnosis and treatment. In the USA, NCC-related hospital charges accounted for more than $908 million from 2003 to 2012 (O’Neal and Flecker, 2015).

The diagnosis of NCC is based on neuroimaging, such as computed tomography (CT) or magnetic resonance imaging (MRI), which should be complemented with serologic testing using enzyme-linked immunoelectrotransfer blot to detect antibodies (White et al., 2018). Based on serial imaging studies, NCC parasites in the parenchymal region of the human brain evolve through 3 distinct phases: (1) the active phase, when the encysted parasite is viable or alive, (2) the degenerative phase, in which the host immune system has targeted the parasite, and after the parasite dies the cyst may completely resolve and be invisible on imaging but, in some cases, (3) it leaves a calcified lesion (Carpio et al., 1994). In extraparenchymal regions, these phases of evolution are less clear and a high proportion of cysts are racemous, in which the cyst looks like a bunch of grapes (Bazan et al., 2016).

The usual treatment of NCC includes anthelmintic drugs to kill the parasite coupled with symptomatic treatment such as antiseizure medication. Albendazole is the recommended anthelmintic drug, although combined therapy with albendazole and praziquantel is recommended for patients with >2 NCC cysts in the brain parenchyma (White et al., 2018). Combined therapy recommendations were initially based on a trial that was stopped early due to demonstrated efficacy (Garcia et al., 2014). Subsequent research, including systematic reviews and additional trials (Dewi et al., 2024; Rani et al., 2024), has reinforced the benefits of combination therapy, particularly its superiority in resolving viable cysts compared to monotherapy. However, continued research is needed to confirm its long-term benefits across diverse patient populations. Albendazole treatment alone only kills about 60% of NCC parasites (Takayanagui and de Haes, 2022), and 38%–49% of patients are free of viable parasites in parenchymal regions by 6 months following treatment, compared to 15%–23% of those receiving placebo (White et al., 2018). Albendazole is less effective for cysts in the extraparenchymal regions of the brain and for degenerating parasites and it has no effect on calcified cysts, which are already dead but can continue to cause symptoms (White et al., 2018).

NCC symptoms are diverse and can range from seizures (occurring in about two thirds of patients) to severe headaches, focal deficits, cognitive deficits and psychiatric symptoms. The clinical manifestations of NCC vary by number, size, location and phase of the parasitic larva and the host’s immune response (Takayanagui, 2001). The overall impact of treatment may vary depending on cyst type, location and associated symptoms. A systematic review and meta-analysis of 7 trials found that those treated with albendazole had significantly lower probability of seizure recurrence than those receiving placebo in only 1 of the 7 studies (Monk et al., 2021). However, 2 other studies not included in the meta-analysis found albendazole associated with a significant decrease in the number of seizures with generalization (generalized seizures or focal seizures with secondary generalization) over follow-up (up to 2 years in 1 study and 5 years in the other) but not in the number of seizures overall or number of partial seizures without generalization (Garcia et al., 2004; Romo et al., 2015). There is no known biological explanation for why albendazole would only impact seizures with generalization, but this may be related to measurement error or reporting bias, especially if non-generalized seizures were under-reported. In addition, the studies found significant associations only when looking at the number of generalized seizures, not the occurrence of any generalized seizures, suggesting lack of statistical power may be a problem. Another study looking at the impact of albendazole on non-seizure symptoms found a significantly lower odds of reporting memory loss and confusion over 2-year post NCC diagnosis, but no treatment benefit for the 7 other symptoms examined (Thapa et al., 2018). However, all these studies followed patients for short periods of time, ranging from 2 to 8 years post-diagnosis. The longer-term symptom profile for NCC has not been described, nor do we know how this profile may vary by treatment, cyst (location and phase) or patient characteristics.

Recent research emphasizes the pivotal role of brain calcifications in NCC, underscoring their potential as a critical factor in the long-term symptomatology of the disease. Calcifications represent the final stage in the life cycle of cysticercosis within the human brain and may cause continued neurological symptoms like seizures (Carpio et al., 2021). The International League Against Epilepsy defines epilepsy as either (1) the occurrence of 2 or more unprovoked seizures (UPS) spaced more than 24 hours apart, or (2) 1 UPS with a probability of subsequent seizures akin to the general recurrence risk (at least 60%) after 2 UPS, within the following decade (Fisher et al., 2014). When considering NCC in this context, the acute symptomatic seizures induced by active and degenerating cysts would not be classified as epilepsy since they are not unprovoked. However, seizures occurring after the complete resolution of NCC cysts would be deemed unprovoked, potentially meeting epilepsy criteria. Whether seizures associated with calcified cysts are provoked or unprovoked is under debate (Carpio et al., 2021). This highlights the importance of assessing the long-term neurological consequences of NCC, especially following parasite death, and how this may relate to the definition of epilepsy.

Therefore, the aims of this study were to (1) describe the long-term symptom profile of NCC patients and (2) examine the impact of albendazole treatment and cyst characteristics (location in the brain and evolutionary phase) at last imaging among participants in a randomized controlled trial (RCT) in Ecuador when contacted 12 years following the end of the RCT. The findings of this study are important to improving understanding around the long-term healthcare needs of NCC patients and can inform the development of more effective therapies for this debilitating condition.

## Methods

### Recruitment and data collection

The data for this study came from an RCT of albendazole treatment for NCC (registered with ClinicalTrials.gov ID #NCT00283699). The study has been described elsewhere (Carpio et al., 2008), but here we provide a brief description. Participants (*n* = 170) were recruited February 2001–2003 from 6 hospitals in Ecuador and were eligible for the study if they presented with new onset symptoms (within past 2 months) suggestive of NCC and were diagnosed with NCC based on CT or MRI with evidence of active and/or degenerating NCC cysts. Patients were not eligible if they had only calcified cysts, were pregnant, had active tuberculosis, syphilis, papilledema, ocular cysticercosis, active ulcers, or any other progressive or life-threatening disorder. Patients who had received treatment for NCC <12 months previously or who had had systemic treatment with steroids ≤30 days since presentation were also ineligible and, halfway through the study, patients with ventricular shunt were excluded for safety reasons.

Study participants were randomized to receive either 400 mg of albendazole or a placebo given orally every 12 hours for 8 days under direct observation. All patients received prednisone 75 mg daily for 8 days, which was then tapered over 2 weeks. For patients weighing less than 50 kg, the dose of albendazole and prednisone were reduced in a standardized manner. Patients also received treatment for symptoms, including antiseizure drugs for those experiencing seizures, which were provided for at least 1 year for patients with a single seizure and at least 2 years for patients who had multiple seizures in the 2 months before study enrolment. The treating neurologist considered medication needed beyond this period on a case-by-case basis. Participants were followed for 2 years (study ending in January 2005) with imaging conducted at baseline, months 1, 6, 12 and 24, from which data were collected on number of NCC cysts in each phase by brain location.

In 2017, 12 years after study completion (14–16 years post NCC diagnosis), we looked at medical records (*n* = 59) or contacted participants (*n* = 17) or family members (*n* = 18) to collect information about symptoms experienced since the trial ended. We extracted all health conditions and symptoms recorded in the post-trial health records.

For participants whose health records were unavailable, we directly contacted the patient and inquired about seizures since the study ended. We then asked an open-ended question about any other health problems experienced since the trial’s conclusion and documented all reported conditions and symptoms. When we were unable to communicate directly with the trial participant, we asked a family member the same questions about the participant’s heath post-trial.

Approval for the original RCT was granted by the Institutional Review Board of Columbia University and the Office for Human Research Protection (OHRP) of the National Institutes of Health in the USA, as well as the ethics committees at each participating hospital. The 12-year post-trial follow-up study was approved by the Ethics Committee at the Hospital Vicente Corral Moscoso of Cuenca, Ecuador.

### Variables examined

The outcome of primary interest was report of any neurological symptom over the 12 years post-trial. We defined neurological symptoms as including seizure, headache, cognitive, sensory and motor dysfunctions, such as disorientation, insomnia, memory difficulties, dementia, language impairment, gait disorder, confusion, depression or other mental health problems, hydrocephalus and vision problems. Secondary outcomes examined included (1) any health problem (neurological and other symptoms), and (2) any specific neurological symptom reported by at least 20 participants, with seizure and headache being the only symptoms that met this criterion. Headache included migraine as well as general headaches (severe and non-severe).

The exposure of primary interest was treatment arm (albendazole vs placebo). We also described the sample in terms of participant characteristics (age at 12-year follow-up and sex), symptoms experienced post-trial and cyst characteristics at last imaging, which occurred 24 months post-diagnosis for 61 participants, 12 months post-diagnosis for 28 participants and 6 months post-diagnosis for 5 participants. Specifically, we looked at presence of cysts in each phase (active, degenerating and calcified) and location (parenchymal only, some extraparenchymal, indeterminate location, or no cysts visible). Extraparenchymal locations included cysts in intraventricular, cisternal and subarachnoid locations, as has been previously described (Montgomery et al., 2019). For standardized regression modelling, we looked at cyst location defined as having any cysts located in extraparenchymal regions of the brain and the presence of any calcified cysts at last imaging. In addition, we described the seizures experienced post-trial by type (focal, including focal aware and focal impaired awareness, generalized and status epilepticus), number of seizures since the end of the trial (1 or more than 1), continuation of antiseizure medication and time between NCC diagnosis and most recent seizure.

### Statistical analysis

We described participant and cyst characteristics from most recent imaging as well as symptoms experienced over the 12-year since trial end overall and by treatment. We also described the seizures experienced during this time period overall and by treatment. We used a Fisher’s exact test to assess the significance of differences in categorical variables by treatment and the Mann–Whitney *U* test for numeric variables.

Using log-binomial regression, we estimated crude risk differences across 4 outcomes, considering treatment, participant age and sex and the presence of extraparenchymal and calcified cysts at last imaging. Due to convergence issues with multivariable binomial regression, we shifted to standardized multivariable logistic regression (Hernan and Robins, 2023). We estimated effects (on a risk ratio scale) for 3 scenarios: (1) albendazole treatment vs placebo, (2) presence of any extraparenchymal cysts and (3) presence of any calcified cysts at last imaging. We adjusted for confounding and informative censoring. All models adjusted for patient age and sex and, for the models looking at cyst characteristics, we also adjusted for treatment arm.

These analyses estimated the risk in hypothetical scenarios where all individuals received the same treatment or had similar cyst characteristics, based on the principles of exchangeability, positivity and consistency to ensure unbiased estimates. Bootstrapping with 1000 samples provided 95% confidence intervals, using RStudio (version 2023.3.0.386), with significance set at *α* = 0.05.

## Results

### Description of participants and persistent symptoms

We collected 12-year post-RCT data on 94 of the 170 participants who completed treatment in the initial trial (55.3%), 49 (52.1%) of whom were in the albendazole arm. A slight majority of participants were male (53.2%), and average age was 55.1 years. At last imaging, participants had an average of 8.1 cysts evident in their brain with 50% having calcified cysts, 40.4% active cysts, 34.3% transitional cysts and 4.3% racemous cysts. Thirty-three percent had cysts in multiple phases. Overall, 27.1% had some cysts in extraparenchymal locations and 11.7% had complete cyst resolution (no NCC cysts visible on last scan).

Over half of participants (52.1 %) reported health problems since the trial ended, with 48.9% reporting 1 or more neurological symptoms, 26.6 % reporting seizures and 22.3% reporting headaches. Other specific symptoms reported by a small number of participants included: gastrointestinal symptoms (4.3%), blood hypertension (3.2%) and myalgia (1.1%). There were no significant differences in participant or NCC characteristics, nor symptoms experienced post-trial by treatment (Table 1).

**Table 1.** Description of study participant, symptom profile over 12 years post-trial and cyst characteristics at last imaging overall and by treatment group

Characteristic	Total	Albendazole	Placebo	*P* value for Fisher’s exact test
Total, *n* (%)	94 (100%)	49 (52.1%)	45 (47.9%)	NA
Sex				1.000
Male, *n* (%)	50 (53.2%)	26 (53.1%)	24 (53.3%)	
Female, *n* (%)	44 (46.8%)	23 (46.9%)	21 (46.7%)	
Patient age in years				0.943^a^
Mean (SD)	55.1 (16.12)	55.1 (16.9)	55.0 (15.5)	
Median (min-max)	54 (18 − 96)	55 (18 − 89)	54 (32 − 96)	
Any symptom over the past 12 years, *n* (%)^b^				0.839
No	45 (47.9%)	24 (49.0%)	21 (46.7%)	
Yes	49 (52.1%)	25 (51.0%)	24 (53.3%)	
Any neurological symptom over the past 12 years, *n* (%)^c^				0.837
No	48 (51.1%)	26 (53.1%)	22 (48.9%)	
Yes	46 (48.9%)	23 (46.9%)	23 (51.1%)	
Any seizure over the past 12 years, *n* (%)				0.648
No	69 (73/4%)	37 (75.5%)	32 (71.1%)	
Yes	25 (26.6%)	12 (24.5%)	13 (28.9%)	
Headache, *n* (%)				0.805
No	73 (77.7%)	39 (79.6%)	34 (75.6%)	
Yes	21 (22.3%)	10 (20.4%)	11 (24.4%)	
Other symptoms reported by a small number of participants				
Gastrointestinal symptoms				0.118
No	90 (95.7%)	45 (91.8%)	45 (100%)	
Yes	4 (4.3%)	4 (8.2%)	0 (0%)	
Hypertension				1.000
No	91 (96.8%)	47 (95.9%)	44 (97.8%)	
Yes	3 (3.2%)	2 (4.1%)	1 (2.2%)	
Myalgia				1.000
No	93 (98.9%)	48 (98.0%)	45 (100%)	
Yes	1 (1.1%)	1 (2.0%)	0 (0%)	
Active cysts				0.058
No	56 (59.6%)	34 (69.4%)	22 (48.9%)	
Yes	38 (40.4%)	15 (30.6%)	23 (51.1%)	
Transitional cysts				1.000
No	62 (66.0%)	32 (65.3%)	30 (66.7%)	
Yes	32 (34.0%)	17 (34.7%)	15 (33.3%)	
Calcified cysts				0.409
No	47 (50.0%)	27 (55.1%)	20 (44.4%)	
Yes	47 (50.0%)	22 (44.8%)	25 (55.6%)	
Racemous cysts				1.000
No	90 (95.7%)	47 (95.9%)	43 (95.6%)	
Yes	4 (4.3%)	2 (4.1%)	2 (4.4%)	
Location of cysts at last imaging, *n* (%)				0.235
Only parenchymal	60 (63.8%)	32 (65.3%)	28 (62.2%)	
Parenchymal and/or extraparenchymal	22 (23.4%)	9 (18.4%)	13 (28.9%)	
Neither (no cysts)	11 (11.7%)	8 (16.3%)	3 (6.7%)	
Location not specified	1 (1.1%)	0 (0%)	1 (2.2%)	

a Mann–Whitney *U* test.

b Any symptom includes neurological symptoms.

c Any neurological symptoms include seizures and headaches.

Among the 11 participants with no cysts visible on last imaging, 3 (27.3%) reported seizures and 1 of these participants also reported headaches (9.1%). Additionally, among the 24 participants with only calcified cysts on their last imaging, 14 (58.33%) reported 1 or more neurological symptoms, including 8 (33.33%) reporting seizures and 9 (37.50%) reporting headaches. No other neurological symptoms and no non-neurological symptoms were reported among those with cyst resolution or only calcified cysts at last imaging. (Data not shown.)

### Seizures experienced during the 12 years post-trial

Among the 25 participants who experienced seizures post-trial, almost all (96%) reported having had only 1 seizure during that time, but 1 participant (4%) had multiple seizures. A majority of those with seizures (64%) were taking antiseizure medication 12-year after the trial ended and no 1 who had not experienced a seizure post-trial was still on antiseizure medication. Time between NCC diagnosis and last seizure was >10 years for 60% of the participants, 5–10 years for 24% and <5 years for 16%. At their last scan, the majority (*n* = 14, 56%) of participants who experienced seizures had active and/or transitional cysts, while 32% (*n* = 8) had only calcified cysts and 12% (*n* = 3) had no cysts (Table 2). Among the 24 participants with only calcifications at last scan, 5/14 (35.7%) of those who received albendazole had seizures while 3/10 (30%) of those who received placebo had seizures. Among those who had active and/or degenerating cysts at last imaging, the numbers were 5/27 (18.5%) with seizures in the albendazole groups and 9/32 (28.1%) in the placebo group. (Data not shown.) Generalized seizures were the most common type (72%), and 1 participant had epileptic status and generalized seizures. Focal seizures were reported by 40% of participants; within this category, focal aware seizures occurred in 32%, focal impaired awareness in 12%, and 1 participant experienced both focal impaired awareness and focal impaired awareness seizures. Three participants reported both focal and generalized seizures (Table 2).

**Table 2.** Description of seizures experienced during the 12 years post-trial

Characteristic	Total	Albendazole	Placebo	*P* value for Fisher’s exact test
Total with seizures	25 (100%)	12 (48.0%)	13 (52.0%)	NA
Number of seizures experienced since end of RCT				0.480
Only one	24 (96%)	11 (91.7%)	13 (100%)	
More than one	1 (4%)	1 (8.3%)	0 (0%)	
Still taking antiseizure medication 12 years post RCT^a^				0.226
Yes	16 (64%)	6 (50%)	10 (76.9%)	
No	9 (36%)	6 (50%)	3 (23.1%)	
Time between NCC diagnosis and last seizure				0.478
<5 years	4 (16%)	1 (8.3%)	3 (23.1%)	
5–10 years	6 (24%)	4 (33.3%)	2 (15.4%)	
>10 years	15 (60%)	7 (58.4%)	8 (61.5%)	
Cyst location				0.435
Had zero cysts at last scan	3 (12%)	2 (16.7%)	1 (7.7%)	
Had only calcified cysts at last scan	8 (32%)	5 (41.65%)	3 (23.1%)	
Had active and/or transitional cysts at last scan	14 (56%)	5 (41.65%)	9 (69.2%)	
Type of seizure				0.278
Focal	10 (40%)	4 (33.3%)	6 (46.2%)	
Focal aware^b^	8 (32%)	4 (33.3%)	4 (30.8%)	
Focal impaired awareness^b^	3 (12%)	0 (0%)	3 (23.1%)	
Generalized^c^	18 (72%)	9 (75%)	9 (69.2%)	
Status epilepticus^d^	1 (4%)	1 (8.3%)	0 (0%)	

a All those taking antiseizure medication during the 12 years post-trial reported having had at least 1 seizure since the end of the trial.

b One participant had both focal aware and focal impaired awareness.

c Three participants reported both focal and generalized seizures.

d One patient with epileptic status also had generalized seizures.

### Association of albendazole treatment with long-term symptoms

In the crude log-binomial model, treatment was associated with a non-significant slightly lower trend in risk for all the long-term symptoms examined (any health symptoms Risk Ratio [RR] = 0.96, *P* = 0.686; any neurological symptom RR = 0.92, *P* = 0.714; seizures RR = 0.85, *P* = 0.752; headaches RR = 0.83, *P* = 0.639) (Table 3). In the standardized multivariable logistic regression models, the results were similar (any health symptom RR = 0.95, *P* = 0.492; any neurological symptoms RR = 0.92, *P* = 0.520; seizures RR = 0.84, *P* = 0.0.501; headaches RR = 0.83, *P* = 0.491) (Table 4).

**Table 3.** Crude log-binomial model results for all 4 outcomes

		Outcome							
		Any symptoms^a^		Any neurological symptoms^b^		Seizure		Headache	
Exposure	*n*	RR (95% CI)	*P* value	RR (95% CI)	*P* value	RR (95% CI)	*P* value	RR (95% CI)	*P* value
Albendazole treatment (ref = placebo)	94	0.96 (0.65–1.41)	0.686	0.92 (0.61–1.39)	0.714	0.85 (0.43–1.66)	0.752	0.83 (0.39–1.77)	0.639
Female (ref = male)	94	1.18 (0.80–1.74)	0.393	1.24 (0.82–1.87)	0.308	1.05 (0.53–2.05)	0.889	1.25 (0.59–2.66)	0.562
Age in years	94	1.01 (1.00–1.02)	0.155	1.00 (0.99–1.01)	0.650	1.01 (0.99–1.03)	0.248	0.99 (0.97–1.02)	0.699
Had extraparenchymal cysts at last imaging (ref = only parenchymal cysts)	82	0.96 (0.62–1.50)	0.865	0.94 (0.60–1.51)	0.792	0.43 (0.14 –1.31)	0.139	0.68 (0.25–1.81)	0.444
Has calcified cysts at last imaging (ref = no calcified cysts)	94	0.96 (0.65–1.41)	0.836	1.09 (0.72–1.65)	0.680	1.08 (0.55–2.12)	0.815	1.62 (0.74–3.55)	0.224

a Any symptom includes neurological symptoms.

b Any neurological symptoms include seizures and headaches.

**Table 4.** Standardized logistic regression model results looking at drug treatment (Albendazole vs Placebo), presence of NCC cysts in extraparenchymal locations and presence of calcified cysts at last imaging as predictors of symptoms experienced by neurocysticercosis patients 14–16 years post-diagnosis

		Outcome							
		Any symptoms^a^		Any neurological symptoms^b^		Seizure		Headache	
Exposure	*n*	RR (95% CI)	*P* value	RR (95% CI)	*P* value	RR (95% CI)	*P* value	RR (95% CI)	*P* value
Albendazole treatment (ref = placebo)^a^	94	0.95 (0.65–1.41)	0.492	0.92 (0.61–1.37)	0.520	0.84 (0.40–1.60)	0.501	0.83 (0.39–1.99)	0.491
Had extraparenchymal cysts at last imaging (ref = only parenchymal cysts)^c^	82	0.91 (0.56–1.52)	0.593	0.91 (0.56–1.52)	0.593	0.37 (0.00–0.90)	0.496	0.69 (0.16–1.91)	0.575
Has calcified cysts at last imaging (ref = no calcified cysts)^d^	94	0.98 (0.63–1.43)	0.654	1.09 (0.70–1.68)	0.484	1.12 (0.52–2.38)	0.488	1.57 (0.70–4.32)	0.529

a Any symptom includes neurological symptoms.

b Any neurological symptoms include seizures and headaches.

c Standardized for participant sex and age at study enrolment.

d Standardized for participant sex, age at study enrolment and treatment (albendazole or placebo).

### Association of cyst location with long-term symptoms

In the crude log-binomial model, having any extra parenchymal cysts at last imaging was associated with a non-significant slightly lower trend in risk of experiencing all symptoms over the 12 years post-trial (any health symptoms RR = 0.96; *P* = 0.865; any neurological symptom RR = 0.94, *P* = 0.792; seizures RR = 0.43, *P* = 0.139; headaches RR = 0.68, *P* = 0.444) (Table 3). In the standardized multivariable logistic regression models, the pattern was similar (any symptom RR = 0.91, *P* = 0.593; any neurological symptoms RR = 0.91, *P* = 0.605; seizures RR = 0.37, *P* = 0.496; headaches RR = 0.69, *P* = 0.575) (Table 4).

### Association of calcified cysts with long-term symptoms

In the crude log-binomial model, having calcified cysts evident at last imaging was associated with a non-significant slightly lower trend in risk of any symptom 12 years post-trial but the association was not statistically significant (RR = 0.96, *P* = 0.836), while those with calcified cysts at last imaging had a non-significant slightly higher trend in risk of all neurological symptoms (any neurological symptom RR = 1.09, *P* = 0.680; seizures RR = 1.08, *P* = 0.815; headaches RR = 1.62, *P* = 0.224) (Table 3). In the standardized multivariable logistic regression models, the associations were similar (any symptom RR = 0.98, *P* = 0.654; any neurological symptoms RR = 1.09, *P* = 0.484; seizures RR = 1.12, *P* = 0.488; headaches RR = 1.57, *P* = 0.529) (Table 4).

## Discussion

Over half of symptomatic NCC patients reported continued health symptoms 2–16 years after their NCC diagnosis, with 48.9% reporting 1 or more neurological symptoms, including 26.6% reporting seizures and 22.3% reporting headaches. Another study focusing on NCC patients in the USA with subarachnoid cysts found 24.2% continued to experience neurological symptoms, including seizures, over a median of 4.2 years post-treatment (Nash et al., 2020). This suggests a high rate of long-term, chronic health problems associated with this infection.

Importantly, there was little difference in the proportion experiencing symptoms by treatment arm, suggesting that treating NCC with anthelminthic medication like albendazole may kill some of the parasites sooner, but it has minimal impact on the symptoms experienced by NCC patients over the long-term. A meta-analysis on the impact of albendazole on symptoms over a shorter follow-up (2–7 years), reported seizure recurrence in 28% of patients (Monk et al., 2021). While 2 studies did find a significant benefit from albendazole on the number of seizures with generalization (Garcia et al., 2004; Romo et al., 2015), in 1 study the benefit declined over time and was only significant between 1 and 12 months post-treatment but not between 13 and 24 months post-treatment (Romo et al., 2015). Thus, any benefit achieved by killing the NCC parasite sooner through anthelminthic drugs likely wears-off quickly as parasites in those not treated die naturally over time and the long-term impact of treatment on the symptoms experienced by NCC patients appears to be minimal. One analysis looking at the impact of albendazole treatment on seizures over time for a 2-year follow-up concluded that the relationship is complex and the direction of the association between albendazole (vs placebo) on seizures changes over time (fewer seizures in the albendazole group over the short term but then more seizures later), with this association mediated by the evolution of the NCC cysts, which is impacted by albendazole (Carpio et al., 2021). Overall, these findings suggest that while albendazole might have some short-term benefits on symptoms, its long-term impact may be limited, and more research is needed to help NCC patients manage and alleviate their symptoms over the long term. The lack of association between long-term symptoms and cyst location (extraparenchymal) and the presence of calcified cysts could also be related to cyst resolution over time and perhaps only imaging results proximate to the time of the symptoms are relevant.

Among the participants who reported having seizures during the 12 years after the trial ended, almost all experienced only 1 seizure, but 64% were still taking antiseizure medication at the time of the follow-up interview, and thus may still be at risk of seizure recurrence. Importantly, 3 (12%) of those experiencing seizures post-trial had zero NCC cysts apparent on their last scan. Thus, these 3 (out of 94, 3.2%) participants may have experienced UPS and be at risk for epilepsy if they experience or have high risk for additional UPS. Some describe NCC as a common cause of epilepsy (Bustos et al., 2021). However, it is also possible that these participants were re-infected after the trial ended or that their classification as having complete cyst resolution was an error (see the limitations section below about misclassification of imaging data). An additional 8 (32%) participants who experienced seizures only had calcified cysts on their last scan. If seizures related to having deceased parasitic calcified cysts can be considered unprovoked remains a question (Carpio et al., 2021). Calcified NCC cysts may continue to emit antigen (Gupta et al., 2002) and perilesional oedema around calcified lesions has been described and may explain continued seizures, although the oedema may also be a result of the seizures (Nash et al., 2008).

The long-term outcomes of NCC patients are not good, with almost half continuing to experience neurological symptoms and 17% still on antiseizure medication 14–16 years after NCC diagnosis. NCC is a neglected infectious disease (CDC, 2022; WHO, 2022a) and this neglect is impacting NCC patients throughout their lifespan. Additional research is needed on how to kill the NCC parasite more effectively and resolve the NCC-related symptoms in the short and long-term.

### Limitations

This study has several limitations to consider when interpreting results. We were only able to collect long-term follow-up data on 55.3% of the participants who completed the trial, indicating high loss to follow-up. This reduces our sample size as well as generalizability as we do not know if the long-term health of those we were unable to get data on was similar to that of participants included in these analyses. It seems likely that those we were able to contact would have a higher prevalence of long-term health problems such that they had continued contact with their doctor, making it more likely that they have up-to-date health record and/or contact information available. This suggests that we overestimated the prevalence of long-term symptoms, but even if we assume that those we were unable to contact had no long-term symptoms, that would still mean that 46/170, or 27.1% of participants experienced neurological symptoms after the trial ended (14.7% seizures and 12.4% headaches), which is in-line with the percent of patients presenting with continued neurological symptoms over the long-term among patients with subarachnoid cysts (24.2%) (Nash et al., 2020) and is still very high. In addition, there was little difference in the proportion of our sample in each treatment arm (49 in the albendazole arm and 45 in the placebo arm), suggesting that randomization was maintained despite high loss to follow-up.

The small sample size limited our ability to run multivariable models with all the covariates of interest. Even with the scaled-back standardized models, some of our non-significant results could be type II errors due to the small sample. In addition, measurement error could be a problem, including misclassification of our outcomes due to misrecall of symptoms experienced over the past 12 years. This misclassification likely led to an underestimation of symptoms experienced since the trial ended as people may forget symptoms experienced a long time ago. It is unlikely that this misclassification was different by treatment, cyst location or presence of calcified cysts at last imaging so it would result in bias towards the null, further reducing our statistical power to identify factors associated with long-term symptoms. But it also means that the high proportion of participants who reported long-term health problems may be an underestimation of the true long-term impact of NCC. The imaging conducted at 24 months included both CT and MRI scans. Calcifications are more detectable on CT scans while active and degenerating cysts are more easily detected on MRIs, suggesting misclassification, but the specific form of misclassification dependent on the type of imaging (Lerner et al., 2012). In addition, inter-reliability measures of radiologist readings of CT and MRI scans tend to be in the mediocre range, for NCC specifically (kappas in 1 study ranging from 0.4 to 0.7 for identifying the presence of any cysts in a specific phase or brain location (Carpio et al., 2008) and for other diseases (e.g. kappas ranging from 0.6 to 0.8 for rating white matter and ischemic lesions (Schryver ELLM et al., 2006) and 0.14–0.78 for early signs of infarction (Wardlaw and Mielke, 2005)). Thus there is potential misclassification of cyst location and phase at last imaging which, again, is likely non-differential by long-term health outcome, resulting in bias towards the null. Furthermore, the last imaging conducted was 2-year post-diagnosis and we really do not know what happened over the subsequent 12 years that might explain some of these symptoms experienced. For example, it is possible that the participants with seizures after the study ended but no NCC cysts apparent at 24 months of imaging experienced reinfection. Additionally, residual confounding is possible due to variables for which we were unable to adjust. Finally, the study findings may not be generalizable to people in other countries or even in Ecuador more recently as changes in healthcare and environment (e.g. reinfection risk) may impact long-term symptoms.

## Conclusion

To our knowledge, this is the longest follow-up of NCC patients to be conducted. Our findings highlight the need for a re-evaluation of current treatment protocols and additional research on how to give NCC patients a symptom-free future following treatment. Given the complexity of NCC pathophysiology and the multifactorial nature of its short- and long-term clinical outcomes, future research should explore more effective treatment, focusing on both parasite mortality and improved patient symptoms, understanding the role of calcified cysts in continued symptoms and conduct longer follow-up with imaging to better understand why many patients continue to experience symptoms over a decade after diagnosis with NCC, indicating that NCC is, in fact, a chronic disease.

## Data Availability

The data are available upon request for valid use to duplicate published papers or answer secondary research questions.

## References

[ref1] Bazan R, Hamamoto Filho PT, Luvizutto GJ, Nunes HR, Odashima NS, Dos Santos AC, Elias Junior J, Zanini MA, Fleury A and Takayanagui OM (2016) Clinical symptoms, imaging features and cyst distribution in the cerebrospinal fluid compartments in patients with extraparenchymal neurocysticercosis. *PLOS Neglected Tropical Diseases* 10(11), e0005115. doi:10.1371/journal.pntd.0005115 27828966 PMC5102378

[ref2] Bustos J, Gonzales I, Saavedra H, Handali S and Garcia HH and Cysticercosis Working Group in P (2021) Neurocysticercosis. A frequent cause of seizures, epilepsy, and other neurological morbidity in most of the world. *Journal of the Neurological Sciences* 427, 117527. doi:10.1016/j.jns.2021.117527 34147957 PMC8800347

[ref3] Carpio A, Kelvin EA, Bagiella E, Leslie D, Leon P, Andrews H, Hauser WA and Ecuadorian Neurocysticercosis G (2008) Effects of albendazole treatment on neurocysticercosis: A randomised controlled trial. *Journal of Neurology, Neurosurg Psychiatry* 79(9), 1050–1055. doi:10.1136/jnnp.2008.144899 18495737

[ref4] Carpio A, Placencia M, Santillan F and Escobar A (1994) A proposal for classification of neurocysticercosis. *Canadian Journal of Neurological Sciences/Journal Canadien Des Sciences Neurologiques* 21(1), 43–47. doi:10.1017/s0317167100048757 8180904

[ref5] Carpio A, Romo ML, Hauser WA and Kelvin EA (2021) New understanding about the relationship among neurocysticercosis, seizures, and epilepsy. *Seizure* 90, 123–129. doi:10.1016/j.seizure.2021.02.019 33632613

[ref6] CDC (1992) Locally acquired neurocysticercosis–North Carolina, Massachusetts, and South Carolina, 1989–1991. *MMWR Morbidity and Mortality Weekly Report* 41(1), 1–4. 1727973

[ref7] CDC (2022) Neglected tropical diseases. https://www.cdc.gov/globalhealth/ntd/diseases/index.html (accessed 28 September 2022).

[ref8] Dewi DAR, Irawati Tjahjo Widuri L, Allatib A, Athallah AA, Balga AI, Arkania N, Nadhira F, Wiliantari NM and Ulfa F (2024) Effectiveness of the antiparasitic combination of albendazole and praziquantel as compared with albendazole monotherapy in the treatment of neurocysticercosis in children: A systematic review and meta-analysis. *Cureus* 16(7), e64617. doi:10.7759/cureus.64617 39149676 PMC11324961

[ref9] Fisher RS, Acevedo C, Arzimanoglou A, Bogacz A, Cross JH, Elger CE, Engel J, Forsgren L, French JA, Glynn M, Hesdorffer DC, Lee BI, Mathern GW, Moshe SL, Perucca E, Scheffer IE, Tomson T, Watanabe M and Wiebe S (2014) ILAE official report: A practical clinical definition of epilepsy. *Epilepsia* 55(4), 475–482. doi:10.1111/epi.12550 24730690

[ref10] Fleury A, Dessein A, Preux PM, Dumas M, Tapia G, Larralde C and Sciutto E (2004) Symptomatic human neurocysticercosis–age, sex and exposure factors relating with disease heterogeneity. *Journal of Neurology* 251(7), 830–837. doi:10.1007/s00415-004-0437-9 15258785

[ref11] Garcia HH, Gonzales I, Lescano AG, Bustos JA, Zimic M, Escalante D, Saavedra H, Gavidia M, Rodriguez L, Najar E, Umeres H and Pretell EJ and Cysticercosis Working Group in P (2014) Efficacy of combined antiparasitic therapy with praziquantel and albendazole for neurocysticercosis: A double-blind, randomised controlled trial. *The Lancet Infectious Diseases* 14(8), 687–695. doi:10.1016/S1473-3099(14)70779-0 24999157 PMC4157934

[ref12] Garcia HH, Pretell EJ, Gilman RH, Martinez SM, Moulton LH, Del Brutto OH, Herrera G, Evans CA and Gonzalez AE and Cysticercosis Working Group in P (2004) A trial of antiparasitic treatment to reduce the rate of seizures due to cerebral cysticercosis. *New England Journal of Medicine* 350(3), 249–258. doi:10.1056/NEJMoa031294 14724304

[ref13] Gupta RK, Kumar R, Chawla S and Pradhan S (2002) Demonstration of scolex within calcified cysticercus cyst: Its possible role in the pathogenesis of perilesional edema. *Epilepsia* 43(12), 1502–1508. doi:10.1046/j.1528-1157.2002.21302.x 12460252

[ref14] Hernan MA and Robins JM (2023) Standardization and the parametric g-formula. In *Causal Inference: What If*. Boca Raton: Chapman & Hall/CRC.

[ref15] Lerner A, Shiroishi M, Zee C, Law M and Go J (2012) Imaging of neurocysticercosis. *Neuroimaging Clinics of North America* 22, 659–676. doi:10.1016/j.nic.2012.05.004 23122261

[ref16] Monk EJM, Abba K and Ranganathan LN (2021) Anthelmintics for people with neurocysticercosis. *Cochrane Database of Systematic Reviews* 6, Article CD000215. doi:10.1002/14651858.CD000215.pub5 PMC8167835 34060667

[ref17] Montgomery MA, Ramos M, Kelvin EA, Carpio A, Jaramillo A, Hauser WA and Zhang H (2019) A longitudinal analysis of albendazole treatment effect on neurocysticercosis cyst evolution using multistate models. *Transactions of the Royal Society of Tropical Medicine and Hygiene* 113(12), 781–788. doi:10.1093/trstmh/trz073 31433058 PMC6903786

[ref18] Nash TE, O’Connell EM, Hammoud DA, Wetzler L, Ware JM and Mahanty S (2020) Natural history of treated subarachnoid neurocysticercosis. *The American Journal of Tropical Medicine and Hygiene* 102(1), 78–89. doi:10.4269/ajtmh.19-0436 31642423 PMC6947806

[ref19] Nash TE, Pretell EJ, Lescano AG, Bustos JA, Gilman RH, Gonzalez AE and Garcia HH and Cysticercosis Working Group in Peru (2008) Perilesional brain oedema and seizure activity in patients with calcified neurocysticercosis: A prospective cohort and nested case-control study. *Lancet Neurology* 7(12), 1099–1105. doi:10.1016/S1474-4422(08)70243-6 18986841 PMC3725597

[ref20] O’Neal SE and Flecker RH (2015) Hospitalization frequency and charges for neurocysticercosis, United States, 2003-2012. *Emerging Infectious Diseases* 21(6), 969–976. doi:10.3201/eid2106.141324 25988221 PMC4451927

[ref21] Rani V, Gehlawat VK and Arya V (2024) Albendazole and praziquantel combination versus albendazole alone in children with multiple neurocysticercosis: An open labelled randomized controlled trial. *Journal of Family Medicine and Primary Care* 13(6), 2300–2304. doi:10.4103/jfmpc.jfmpc_733_23 PMC11254057 39027868

[ref22] Romo ML, Wyka K, Carpio A, Leslie D, Andrews H, Bagiella E, Hauser WA, Kelvin EA and Ecuadorian Neurocysticercosis G (2015) The effect of albendazole treatment on seizure outcomes in patients with symptomatic neurocysticercosis. *Transactions of the Royal Society of Tropical Medicine and Hygiene* 109(11), 738–746. doi:10.1093/trstmh/trv078 26433183

[ref23] Savioli L (2010) *First WHO Report on Neglected Tropical Diseases: Working to Overcome the Global Impact of Neglected Tropical Diseases*. World Health Organisation, 1–169.

[ref24] Schantz PM, Moore AC, Munoz JL, Hartman BJ, Schaefer JA, Aron AM, Persaud D, Sarti E, Wilson M and Flisser A (1992) Neurocysticercosis in an Orthodox Jewish community in New York City. *New England Journal of Medicine* 327(10), 692–695. doi:10.1056/NEJM199209033271004 1495521

[ref25] Schryver ELLM D, van Gijn J, Kappelle L, Koudstaal P and Algra A ESPRIT Study Group (2006) Severity of cerebral white matter lesions and infarcts in patients with transient or moderately disabling cerebral ischaemia: Reproducibility of grading by neurologists. *European Journal of Neurology* 13,901–903. 16879303 10.1111/j.1468-1331.2006.01269.x

[ref26] Takayanagui OM (2001) Neurocysticercosis. *Revista da Sociedade Brasileira de Medicina Tropical* 34(3), 283–290. doi:10.1590/S0037-86822001000300010 11460216

[ref27] Takayanagui OM and de Haes TM (2022) Update on the diagnosis and management of neurocysticercosis. *Arquivos de Neuro-Psiquiatria* 80(5 Suppl 1). doi:10.1590/0004-282X-ANP-2022-S115 PMC9491409 35976305

[ref28] Thapa K, Romo ML, Carpio A, Leslie D, Andrews H, Hauser WA and Kelvin EA (2018) The effect of albendazole treatment on non-seizure outcomes in patients with symptomatic neurocysticercosis. *Transactions of the Royal Society of Tropical Medicine and Hygiene* 112(2), 73–80. doi:10.1093/trstmh/try023 29579308 PMC6019022

[ref29] Wallin MT and Kurtzke JF (2004) Neurocysticercosis in the United States: Review of an important emerging infection. *Neurology* 63(9), 1559–1564. doi:10.1212/01.wnl.0000142979.98182.ff 15534236

[ref30] Wardlaw JM and Mielke O (2005) Early signs of brain infarction at CT: Observer reliability and outcome after thrombolytic treatment—systematic review. *Radiology* 235, 444–453. doi:10.1148/radiol.2352040262 15858087

[ref31] White AC,J, Coyle CM, Rajshekhar V, Singh G, Hauser WA, Mohanty A, Garcia HH and Nash TE (2018) Diagnosis and treatment of neurocysticercosis: 2017 clinical practice guidelines by the Infectious Diseases Society of America (IDSA) and the American Society of Tropical Medicine and Hygiene (ASTMH). *Clinical Infectious Diseases* 66(8), e49–e75. doi:10.1093/cid/cix1084 29481580 PMC6248812

[ref32] WHO (2022a) Negletect tropical diseases. https://www.who.int/health-topics/neglected-tropical-diseases#tab=tab_1 (accessed 28 September 2022).

[ref33] WHO (2022b) Taeniasis/cysticercosis. https://www.who.int/news-room/fact-sheets/detail/taeniasis-cysticercosis (accessed 15 October 2022).

